# Screening for asymptomatic deep vein thrombosis in COVID-19 patients admitted to the medical ward: a cross-sectional study

**DOI:** 10.1007/s40477-022-00689-w

**Published:** 2022-05-14

**Authors:** T. J. Olgers, A. W. E. Lieveld, B. Kok, J. Heijmans, D. Salet, N. L. Assman, D. F. Postma, M. P. Bauer, P. W. B. Nanayakkara, K. Meijer, F. H. Bosch, H. Kooistra

**Affiliations:** 1 Department of Internal Medicine, Section Acute Internal Medicine, University of Groningen, University Medical Center Groningen, Hanzeplein 1, 970 RD Groningen, The Netherlands; 2 General and Acute Internal Medicine Department of Internal Medicine, Amsterdam Public Health Research Institute, Amsterdam University Medical Center, Amsterdam, The Netherlands; 3 Section Acute Internal Medicine, Department of Internal Medicine, Radboud University Medical Center, Nijmegen, The Netherlands; 4 Department of Internal Medicine, Infectious Diseases Service, University of Groningen, University Medical Center Groningen, Groningen, The Netherlands; 5 Department of Internal Medicine, Leiden University Medical Center, Leiden, The Netherlands; 6 Department of Haematology, University of Groningen, University Medical Center Groningen, Groningen, The Netherlands

**Keywords:** Asymptomatic DVT, COVID-19, POCUS, Ward

## Abstract

**Purpose:**

Patients with COVID-19 have an increased risk for venous thrombo-embolism (VTE), especially pulmonary embolism. The exact prevalence of asymptomatic DVT is not known, as is the usefulness of screening for DVT in patients admitted to ward with COVID-19. We have studied the prevalence of asymptomatic DVT.

**Methods:**

We performed a cross-sectional observational multi-center study at four university medical centers in The Netherlands. All adult patients admitted with COVID-19 to a medical ward were eligible for inclusion, including patients who were transferred back from the ICU to the ward. The study protocol consisted of weekly cross-sectional rounds of compression ultrasound.

**Results:**

In total, 125 patients were included in the study. A significant proportion of patients (*N* = 34 (27%)) had developed a VTE during their admission for COVID-19 before the study ultrasound was performed. In most VTE cases (*N* = 27 (79%)) this concerned pulmonary embolism. A new asymptomatic DVT was found in 5 of 125 patients (4.0%; 95% CI 1.3–9.1%) (Table 2). Nine patients (7.2%; 95% CI 3.3–13.2%) developed a VTE (all PE) diagnosed within 28 days after the screening US was performed.

**Conclusion:**

We have shown a low prevalence (4%) of newly discovered asymptomatic DVT outside the ICU-setting in COVID-19 patients. Despite this low prevalence, nine patients developed PE (7%) within 28 days after ultrasound. This favors the hypothesis of local thrombus formation in the lungs. Based on our findings and literature, we do not recommend US-screening of asymptomatic patients with COVID-19 admitted to the ward.

## Introduction

In December 2019, severe acute respiratory syndrome coronavirus 2 (SARS-CoV-2) emerged [1]. The clinical spectrum of this pathogen ranges from an asymptomatic infection and mild respiratory disease to severe viral pneumonia, collectively called coronavirus disease 2019 (COVID-19) [2]. These latter patients have higher levels of D-dimer compared to non-severe cases. A high D-dimer reflects coagulopathy and disseminated intravascular coagulation (DIC) and is associated with an increased risk of death [3].

Studies on COVID-19 patients show a marked increase in the risk of venous thromboembolism (VTE). Pulmonary embolism (PE) occurred in 20.4% of French patients admitted to the intensive care unit (ICU) despite prophylactic antithrombotic treatment in almost all affected patients (20/22) [4]. In a Dutch single-center cohort study, VTE was observed in 29 of 74 ICU patients despite thrombosis prophylaxis [5]. A multicenter study showed a 31% incidence of any thromboembolism (arterial and/or venous) in ICU patients despite standard prophylactic anticoagulation use, in most cases pulmonary embolism (81% of total thrombotic events) [6]. These studies highlight the problem of thrombosis despite thromboprophylaxis in the ICU.

The incidence of (a)symptomatic deep-vein thrombosis (DVT) outside the ICU is much lower. A study from Italy showed an incidence of VTE of 3.8% for patients admitted to the general ward, and half of them occurred within 24 h of admission [7]. This is in line with the previously mentioned Dutch study, which showed an incidence of symptomatic DVT in 3% (4 of 124) of patients admitted to the ward, and asymptomatic DVT was only found in ICU patients [5]. However, data on asymptomatic VTE during ward admissions are scarce, as only 17 of these patients were screened with an ultrasound. It is unknown if PE comes from DVT or reflects a local pulmonary process of inflammation and coagulation in COVID-19. Whether early detection of asymptomatic DVT provides the opportunity to increase anticoagulation and prevent clinical deterioration due to PE in patients admitted to medical wards needs further research. Also, optimal prophylactic anticoagulation dose for patients admitted to the ward with COVID-19 is currently highly discussed [8].

We conducted a study to determine the prevalence of asymptomatic DVT in COVID-19 patients admitted to medical wards. Our hypothesis is that COVID-19-related VTE is predominantly caused by local inflammation in the lungs and not the result of dislocating clots from DVT. As such, we expect a low prevalence of asymptomatic DVT.

## Methods

We performed a cross-sectional observational multi-center study at four university medical centers in the Netherlands. All adult patients admitted with COVID-19 to a medical ward were eligible for inclusion, including patients who were transferred back from the ICU to the ward. The inclusion started the 27th of April 2020 and ended the 19th of June 2020 when the first wave of COVID-19 in the Netherlands ended. Patients were considered to have COVID-19 if this was confirmed with a positive PCR-test or when the treating (multidisciplinary) medical team concluded that it was clinically and radiographically highly suspected.

The study protocol consisted of weekly cross-sectional rounds of compression ultrasound instead of examining every consecutive patient to save scarce personal protection equipment. Every patient was examined only once. Extended compression ultrasonography (ECUS) was performed on both legs with appreciation of the femoral vein and popliteal vein for at least five standardized points: the common femoral vein, the femoral vein at the entrance of the greater saphenous vein, the femoral vein at the branching of the lateral perforator vein, the distal femoral vein, and the popliteal vein. Distal DVT was not part of the diagnosis of DVT, as the clinical significance and need for anticoagulation in distal DVT is unsure. Thrombosis was defined as the inability to completely compress the venous lumen.

The ultrasound exam was stored in the patient’s record. When DVT was diagnosed, the treating physician was informed promptly. Subsequent treatment of (asymptomatic) VTE was not part of the research protocol and depended on local guidelines and/or as indicated by the treating physician. Routine baseline characteristics were documented, including the use of anticoagulants (prophylactic and therapeutic dose) and previous VTE. The primary outcome was the finding of asymptomatic DVT.

### Statistical analysis

Patient characteristics were analyzed with descriptive statistics. The proportion of VTE was assessed. Descriptive analysis was performed with SPSS 23.0. Based on a prevalence of asymptomatic DVT of 2.5% and a confidence interval of 0–5%, 150 patients would be needed for inclusion.

## Results

In total, 144 patients were screened for eligibility; 17 patients were excluded because of lack of informed consent. Another two patients were already discharged before scanning was possible. The remaining 125 patients were included in the study. Baseline characteristics are shown in Table 1.

**Table 1:** Baseline characteristics

	All patients (*N* = 125)	Patients with DVT at US screening (*N* = 5)
Baseline characteristics		
No of hospitals	4	2
Male sex—no (%)	89 (71)	4 (80)
Age—median (IQR)	62 (53–70)	49 (37–67)
BMI—median (IQR)	27.0 (24.0–30.7)	26.8 (23.9–31.1)
Reason of admission—no (%)		
Suspected or proven COVID	122 (98)	5 (100)
PCR sars-cov2—no (%)		
Positive	122 (98)	5 (100)
Negative but clinically and radiographically highly suspected	3 (2)	0 (0)
At admission history of—no (%)		
VTE	9 (7)	0 (0)
Currently active malignancy	6 (5)	0 (0)
Use of thromboprophylaxis—no (%)		
No	5 (4)	1 (20)
Low prophylactic dose	36 (29)	0 (0)
High prophylactic dose	27 (22)	1 (20)
High (therapeutic) dose	57 (46)	3 (60)
At day of ultrasound		
Days of COVID-symptoms—median (IQR)	25 (13–38)	46 (24–75)
Days of admission—median (IQR)	13 (5–28)	39 (10–58)
Prior ICU admission—no (%)	64 (51)	4 (80)
Days of prior ICU-admission—median (IQR)	20 (12–29)	38 (18–48)
DVT related symptoms—no (%)	0 (0)	0 (0)
VTE during current admission before US—no (%)	34 (27)	3 (60)
Pulmonary embolism	27 (22)	2 (40)
DVT leg	5 (4)	0 (0)
DVT arm	2 (2)	1 (20)

Data are presented as absolute numbers with (%) or interquartile range (IQR)

*ICU* intensive care unit, *VTE* venous thromboembolism, *DVT* deep vein thrombosis, *US* ultrasound

COVID-19 was proven with PCR in 122/125 patients (98%). A history of VTE before admission was present in 9 patients (7%). Half of included patients had a prior ICU admission.

A significant portion of patients (*N* = 34 (27%)) had developed a VTE during their admission for COVID-19 before the study ultrasound was performed, mostly pulmonary embolism (*N* = 27 of 34 (79%)). DVT of the arm and DVT of the leg were present in two and five patients respectively.

The majority of patients received low molecular weight heparin when the screening ultrasound was performed. This included a prophylactic dose in 63 patients (51%)) and a high (therapeutic) dose in 57 patients (46%). Only five patients received no pharmacological thromboprophylaxis. In half of the patients, the ultrasound was performed after intensive care admission, with a median ICU length-of-stay of 20 days. There were no patients with symptoms of deep-vein thrombosis. The prevalence of asymptomatic deep-vein thrombosis of the leg was still four of 64 patients (6.3%) in the subgroup of patients recovering from ICU admission (51%). One patient admitted to the ward without prior ICU admission showed a DVT (1.6%).

### Asymptomatic deep-vein thrombosis

A new asymptomatic DVT was found in five of 125 patients (4.0%; 95% CI 1.3–9.1%) (Table 2).

**Table 2:** Prevalence of DVT

	All patients *N* = 125 *N* (%; 95% CI)	Patients with previous VTE (during admission) *N* = 34 *N* (%; 95% CI)	Patient without previous VTE during admission *N* = 91 *N* (%; 95% CI)
Outcome			
Asymptomatic DVT found with US-screening	5 (4.0%; 1.3–9.1%)	3 (8.8%; 1.9–23.7%)	2 (2.2%; 0.3–7.7%)
VTE diagnosed within 28 days after study ultrasound	9 (7.2%; 3.3–13.2%)	1 (2.9%; 0.1–15.3%)	8 (8.8%; 3.9–16.6%)
Pulmonary embolism	9 (7.2%; 3.3–13.2%)	1 (2.9%; 0.1–15.3%)	8 (8.8%; 3.9–16.6%)
DVT leg	0 (0.0%; 0.0–2.9%)	0 (0%; 0.0–10.3%)	0 (0%; 0.0–4.0%)
DVT arm	0 (0.0%; 0.0–2.9%)	0 (0%; 0.0–10.3%)	0 (0%; 0.0–4.0%)
Days after study ultrasound—median (IQR)	3 (0.5–7.5)	0 (N/A)	3.5 (1.3 -7.8)
30 day mortality	10 (8.0%; 3.9–14.2%)	1 (2.9%; 0.1–15.3%)	8 (8.8%; 3.9–16.6%)

Data are presented as absolute numbers with (%), interquartile range (IQR) or 95% confidence intervals (95% CI)

*US* ultrasound, *VTE* venous thromboembolism, *DVT* deep vein thrombosis

In only two patients (1.6%; 95% CI 0.2–5.7%) therapeutic anticoagulant treatment was initiated. The remaining three patients were already therapeutically anticoagulated for a prior VTE. One patient received intermediate-dose thromboprophylaxis, and the final patient had no anticoagulation. Nine patients (7.2%; 95% CI 3.3–13.2%)) had a VTE diagnosed within 28 days after the screening ultrasound (US) was performed with a median of three days (IQR 0.5–7.5). In all cases, the VTE concerned PE, and no DVT was diagnosed. The overall 30-day mortality was 8.0% (95% CI 3.9–14.2%) in all patients, and one out of five patients with an asymptomatic DVT died (20%).

## Discussion

### Prevalence of asymptomatic DVT

Our study shows a prevalence of 4% (95% CI 1.3–9.1%) of asymptomatic proximal DVT in patients hospitalized with COVID-19 outside the ICU. Half of these patients were already treated with therapeutic-dose anticoagulation because of previously diagnosed VTE during their admission. Finding an asymptomatic deep-vein thrombosis in these patients had no clinical or therapeutic consequence. Despite a negative screening US, nine of 125 (7.2%) patients developed PE after the US exam (with a median of three days).

Our study is in line with findings in a previous Dutch study, as they showed a low incidence of symptomatic DVT (1.6%) and asymptomatic DVT (none of the 17 patients) of patients with COVID-19 on a general ward, in contrast to a cumulative incidence of 32% in ICU patients [5]. A study from Spain included 156 patients with COVID-19 [9]. The ultrasound for asymptomatic DVT was positive in 23 patients (14.7%), but in 22 patients it only concerned a distal DVT. Also, they included patients with an elevated D-dimer (> 1000 ng/mL)—these patients had to be hospitalized for at least 48 h—and patients were excluded if therapeutic doses of anticoagulation were used, limiting the generalizability of their findings. No patient with a negative ultrasound developed PE at the time of the study’s closure. An Italian study included 84 hospitalized COVID-19 patients and showed an overall proximal DVT incidence of 2.4% [10]. They were not able to report clinical outcomes of patients with (distal) DVT compared to subjects without DVT.

In another small study from Italy, 66 patients who were hospitalized for at least 5 days for COVID-19 underwent 2-points compressive ultrasonography in a single day [11]. The presence of an asymptomatic proximal DVT was confirmed in nine patients (13.6%), which was bilateral in three patients. Additional scanning in patients showed PE in five of them. Almost all patients received standard-dose thromboprophylaxis. Finally, a small retrospective French study of 42 patients reported a cumulative incidence of asymptomatic DVT of 19% (in eight of 42 patients) [12]. However, in seven out of eight cases, it was only a distal DVT giving an incidence of proximal DVT of 2.3%. More recent studies confirm the low prevalence of asymptomatic proximal DVT [13, 14]. In conclusion, all besides one study showed a low incidence of proximal DVT outside the ICU. Therefore, screening for asymptomatic DVT on a general ward does not seem useful [15].

### Underlying pathophysiology

The low prevalence of DVT in contrast to relatively high rates of PE, even after ICU discharge, may suggest local pulmonary thrombus formation and unique characteristics of COVID-19 coagulopathy. This theory is supported by several studies showing that multiple factors are involved in COVID-19-associated coagulopathy with high rates of venous thromboembolism, although the exact mechanisms are yet to be elucidated [16–18]. The complex thromboinflammatory process in COVID-19 include endotheliopathy due to direct endothelial infection with SARS-COV-2 and indirect inflammation. The elevated levels of clotting factors and the loss of thromboprotective function with glycocalyx damage also contribute to the coagulopathy with local micro- and macro-vascular thrombosis.

### Limitations

First, ECUS is a full ultrasound exam of the deep venous system but does not encompass isolated calf thrombosis. These may have been missed and may progress to deep-vein thrombosis, but optimal treatment of isolated calf thrombosis, including anticoagulant regimes, are still being discussed. Second, we stopped the study after 125 inclusions, 25 patients short of our calculated sample size, due to a lack of COVID-19 patients in the summer of 2020. However, we do not think this had a large influence on our study results. Furthermore, the majority already received an intermediate dose or therapeutic anticoagulation. Lastly, we have only performed an ultrasound once in each patient, and later development of asymptomatic DVT was not registered. However, no patient developed a symptomatic DVT within 28 days.

## Conclusion

We have shown a low prevalence (4%) of newly discovered asymptomatic DVT outside the ICU setting in COVID-19 patients. Sixty percent of those patients were already treated with a therapeutic dose of anticoagulants because of a symptomatic thrombosis at another site. No patients developed a new DVT within 28 days after the ultrasound, but nine patients did develop PE (7%). This may be explained by a local process of thrombus formation in the lungs instead of a dislocating thrombus from the extremities. Based on our findings and the extant literature, we do not recommend US screening of asymptomatic patients with COVID-19 admitted to the ward.
